# A Personalized Multidisciplinary Approach to Evaluating and Treating Autism Spectrum Disorder

**DOI:** 10.3390/jpm12030464

**Published:** 2022-03-14

**Authors:** Richard E. Frye

**Affiliations:** 1 Department of Child Health, College of Medicine—Phoenix, University of Arizona, Phoenix, AZ 85004, USA; rfrye@phoenixchildrens.com; Tel.: +1-602-933-1100; 2 Section on Neurodevelopmental Disorders, Barrow Neurological Institute, Phoenix Children’s Hospital, Phoenix, AZ 85016, USA

**Keywords:** autism spectrum disorder, environment, multidisciplinary, optimal outcomes

## Abstract

Autism Spectrum Disorder (ASD) is a complex neurodevelopmental disorder without a known cure. Current standard-of-care treatments focus on addressing core symptoms directly but have provided limited benefits. In many cases, individuals with ASD have abnormalities in multiple organs, including the brain, immune and gastrointestinal system, and multiple physiological systems including redox and metabolic systems. Additionally, multiple aspects of the environment can adversely affect children with ASD including the sensory environment, psychosocial stress, dietary limitations and exposures to allergens and toxicants. Although it is not clear whether these medical abnormalities and environmental factors are related to the etiology of ASD, there is evidence that many of these factors can modulate ASD symptoms, making them a potential treatment target for improving core and associated ASD-related symptoms and improving functional limitation. Additionally, addressing underlying biological disturbances that drive pathophysiology has the potential to be disease modifying. This article describes a systematic approach using clinical history and biomarkers to personalize medical treatment for children with ASD. This approach is medically comprehensive, making it attractive for a multidisciplinary approach. By concentrating on treatable conditions in ASD, it is possible to improve functional ability and quality of life, thus providing optimal outcomes.

## 1. Introduction

Autism spectrum disorder (ASD) is a behaviorally defined disorder [1] with the most recent Center for Disease Control and Prevention estimates suggesting that it affects 1 in 44 children in the United States (US) [2]. The gold-standard therapy for core symptoms of ASD at the current time is behavioral therapy, which is most effective if started early in life [3,4]. Unfortunately, despite implementation of early intensive behavioral therapies combined with educational approaches, only a minority of children obtain optimal outcomes [5,6] and most individuals with ASD require life-long supportive care [7]. The economic burden of intense and continuous educational, medical and social support is impressive [8] with the lifetime social costs to date in the US estimated to be more than $7 trillion [9]. In addition, the disability of a child creates a spillover effect, decreasing the quality of life for the entire family [10,11,12]. Given the longitudinal data over the last 20 years from the Autism and Developmental Disabilities Monitoring Network, which suggests that the prevalence of ASD continues to rise despite efforts to recognize and treat it early, ASD may be one of the more significant diseases of our lifetime.

One factor that has limited the success of the treatment of children with ASD in reaching optimal outcomes is the fact that there is still a lack of a clear understanding of the underlying biology that drives the behavioral phenotype of ASD. There are several reasons that research into the underlying biology is difficult. First, ASD is very heterogenous, with many different phenotypical clinical presentations and likely many underlying causes. Second, the underlying pathophysiology is poorly understood, with new trends emerging despite decades of research. Indeed, physiological systems such as redox and mitochondrial metabolism as well as the immune system appear to also play a prominent role in ASD, but an understanding of the mechanism in which they cause disease is still emerging [1]. The fact that there is high heritability within families has driven the search for a genetic cause for ASD, but the empirical evidence suggests that the etiology is much more complex than a simple Mendelian inherited disorder [13]. Evidence now points to the importance of environmental–genetic interactions playing a prominent role in the etiology of ASD [14], particularly the prenatal environment [15]. Third, it is becoming clear that many children with ASD are medically complex [16,17]. Thus, a comprehensive approach is needed to identify and treat conditions that can improve the quality of life and promote optimal outcomes.

## 2. A Comprehensive Medical View of Autism Spectrum Disorder

### 2.1. Differentiating Medical Symptoms and Autism Spectrum Disorder Symptoms

To better understand ASD and its associated symptoms, we need to look at its definition. ASD is defined by specific core symptoms. The current diagnostic criterion is defined by the Diagnostic Statistical Manual of Mental Disorders Version 5 (DSM-5), where ASD is defined as one disorder, without subcategories but with three levels of severity for each of the two core symptom categories (Table 1). Core symptoms fall into two major categories—Social-Communication Impairment and Restricted, Repetitive Behavior; symptoms are necessary in both categories to meet diagnostic criteria for ASD.

Most importantly, the core symptoms of ASD need to be severe enough to affect an individual’s ability to function such that they require external supports or accommodations in order to function in everyday life. Specifically, not only does an individual need to have the core symptoms of ASD, but also the symptoms need to interfere with their life to the extent that limits functional abilities. This is an important point, because if it is possible through treatment to reduce the interference of symptoms with everyday life, then, at least theoretically, an individual would no longer meet criteria for ASD because their symptoms, although present, do not limit their ability to function.

Separate from these core symptoms are several specific and non-specific specifiers to further describe common related and co-occurring conditions. Related to the ASD diagnosis are many specific associated behaviors. Although the core ASD symptoms and associated behaviors are common targets of treatments, such symptoms are modulated by co-occurring conditions outlined in the specifiers, but such conditions are less commonly addressed even though addressing them could have a profound effect on the core ASD symptoms and associated behaviors.

In fact, by addressing these associated symptoms and allowing an individual to substantially function better, the severity of the disorder might be minimized and the diagnosis itself might be eliminated. In this sense, the goal of any treatment may not be to “cure” an individual of ASD, but rather reduce the symptoms so an individual no longer requires supports for their symptoms. Specifically, the severity of the core symptoms of ASD are rated on a scale from 1 to 3 based on the amount of support that is required for each core symptom category (Social Communication; Repetitive and Restricted Behaviors and Interests): Level 1: Requiring support because of the symptoms; Level 2: Requiring substantial support because of the symptoms; Level 3: Requiring very substantial support because of the symptoms. Thus, the goal of treatment may be not to completely eliminate symptoms but rather to reduce the symptoms to the level where no support is needed, essentially a Level 0. Thus, this manuscript outlines how we might reduce both core and associated symptoms together to improve the function of the individual with ASD as to eliminate the need for supports.

We believe that there are many medical and environmental factors that are associated with ASD, which we will describe in detail below. Although it is not clear whether medical disorders and environmental factors are the cause of ASD, it is becoming clear that medical disorders and environmental factors modulate ASD symptoms. Thus, it is important to carefully and quantitatively reassess ASD, behavioral and medical symptoms as treatment is ongoing to ensure that the target ASD symptoms are being adequately addressed.

### 2.2. Both Organ Specific and Systemic Medical Disorders Are Associated with Autism Spectrum Disorder

**Figure 1:**
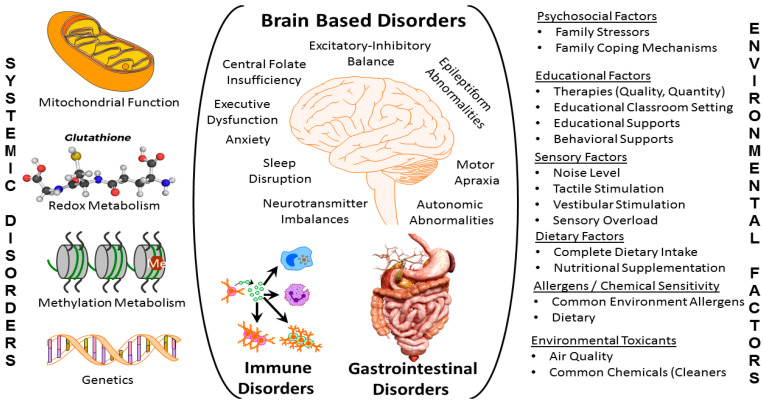
Potentially treatable abnormalities associated with Autism Spectrum Disorders (ASD). Overview of the multiple medical and associated potentially identifiably and treatable abnormalities associated with ASD. The center represents the specific organs commonly affected in ASD while the left column depicts the systematic medical abnormalities that may have widespread effects on multiple organs. The column on the right demonstrates multiple environmental factors that may modulate the severity of the underlying medical abnormalities.

Behavior is believed to originate from the brain, but evidence has emerged showing other organ systems in addition to the brain to be associated with ASD and influence ASD symptoms. For example, recent studies estimate that over 95% of children with ASD have at least one comorbid medical diagnosis [16]. In fact, a recent study which analyzed medical claim data found three patterns of comorbid conditions in children with ASD [17]. For about 50% of the children, the numbers of comorbid conditions were low and they occurred with similar prevalence to the general population. For about 27% of children with ASD, a medium number of comorbid conditions occurred with developmental delays and auditory conditions being most prevalent. Lastly, about 23% of children with ASD demonstrated many comorbid conditions, with immune, gastrointestinal (GI) and psychiatric conditions being most prevalent. Although these data suggest that there is one group of very medically complex children with ASD that require special attention, such a conclusion would underappreciate that common medical conditions of childhood can result in significant exacerbation to ASD symptom in children with ASD making even common medical conditions important to address aggressively. In fact, a recent framework has suggested that children with ASD should be sub-grouped based on their response to treatment of comorbid conditions [18]. Furthermore, studies have suggested that improving healthcare provider awareness of these comorbid and co-occurring conditions can improve the quality of care for children with ASD [19]. Figure 1 outlines some of the organ-based conditions (center), systematic disturbances (left) as well as environmental factors (right) which can exacerbate ASD symptoms.

Environmental factors (Figure 1, right) including socioeconomic [20,21], psychosocial [22] (including parenting competence [20] and involvement [23]), educational [24,25] (including the positivity of the relationship between student and teacher [26]), sensory [27], dietary [28], allergens, chemicals and toxins [29], are common and widespread, meaning that efforts to address these factors involve intervention into everyday life in multiple settings. Organ based factors (Figure 1, middle) include the brain, GI and immune systems, although other systems may be involved. Abnormalities in these organ systems usually, but not always, can be determined from reviewing symptomology as well as performing biomarker and diagnostic testing. Many systematic abnormalities in physiology (Figure 1, left), particularly with respect to mitochondrial, redox, folate and cobalamin metabolism, are associated with ASD. Such systemic abnormalities are particularly important to assess as abnormalities in both the brain and other organ systems can be simultaneously affected, compounding the severity of the disorder. Unfortunately, these latter systemic abnormalities are under-researched and the biomarkers which represent such pathophysiological processes are underdeveloped, making their management limited to highly trained experts.

### 2.3. An Approach to Comprehensive and Systematic Evaluation and Treatment of Autism Spectrum Disorder

As children with ASD may be complex, with potentially many medical and environmental conditions driving their behavior, it is of the utmost importance to take a systematic approach to evaluating and treating their symptoms. Given that many conditions could be driving multiple symptoms, it would behoove the treating clinician to ensure that the evaluation is as comprehensive as possible. Furthermore, because of the complexity it is important to address one treatment target at a time to determine if a positive (or possibly negative) response occurs. This could be a specific targeted symptom or a specific underlying abnormality that is identified. Once one target is addressed, then the next can be addressed in an iterative fashion.

**Figure 2:**
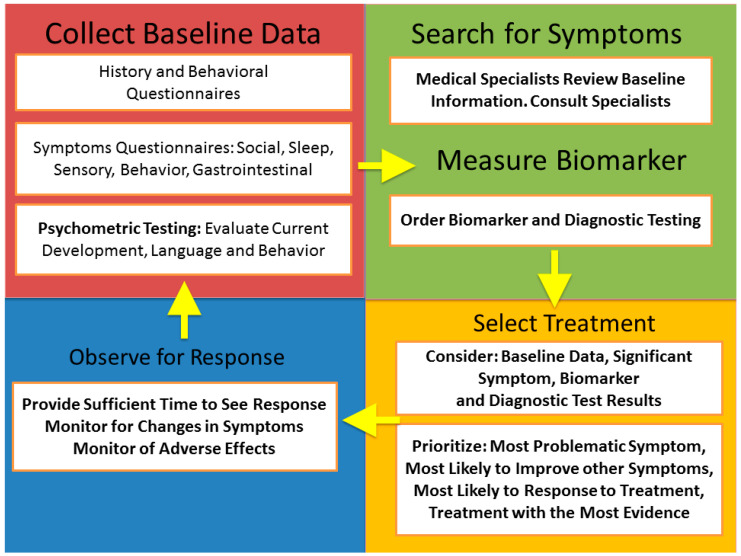
BaS-BiSTOR (collect Baseline data, search for Symptoms, measure Biomarkers, Select Treatment, Observe for Response) flowchart.

This processes of iterative evaluation and treatment is outlined in Figure 2 and referred to by the acronym BaS-BiSTOR (collect Baseline data, search for Symptoms, measure Biomarkers, Select Treatment, Observe for Response). First, it is important to collect good baseline data with a comprehensive evaluation so that there is a clear understanding of the underlying symptoms and so that the progress throughout the process can be continually monitored at each iteration. Second, the baseline data need to be considered and further symptomatology data, specialty diagnostic evaluations and biomarker data need to be obtained. Third, once the detailed evaluations have been conducted, a treatment target must be selected. There are several factors that must go into prioritizing the treatment. While it is important to target the most problematic symptoms, it is also important to target the process that is most likely to have the widest effect on the most symptoms. Additionally, it is often important to start with the treatment that is most likely to be successful based on the individual patient. A successful treatment can motivate a family to continue the approach of treating underlying abnormalities and continue prescribed treatments, whereas a lot of the time, failure will lead to a loss in follow-up. Lastly, it is of the utmost importance that the treatment chosen is based on evidence-based studies. Although treatments with less documented evidence may be used, they should be used as a last resort. Fourth, it is important to give the treatment an appropriate time to work while monitoring the patients’ progress. Once enough of a trial period has elapses, the treatment is either continued or discontinued and the process of obtaining a new baseline and consideration of a new treatment is repeated until function is optimal. Of course, the new baseline assessment at each re-evaluation may not need to be as extensive as the initial baseline assessment.

## 3. Specific Factors to Address in Autism Spectrum Disorder

Here, we outline the evidence for the relationship between symptom severity and specific areas of abnormalities and their association with ASD, as well as discuss the potential assessment tools (Table 2) and provide examples of treatable conditions. This is not meant to be an exhaustive list as the field is moving and changing very quickly. Rather, this is meant to provide a framework.

### 3.1. Evaluating Core Symptoms in Autism Spectrum Disorder

First, it is important to confirm the diagnosis of ASD and indicate the severity of ASD symptoms. Although the exact evaluations used for ASD diagnosis vary, the gold-standard is considered either the Autism Diagnostic Observation Schedule (ADOS) and/or the Autism Diagnostic Interview–Revised (ADI–R), which require highly experienced raters [30]. Such diagnostic assessments are usually performed to establish a diagnosis and are not typically repeated often, so it is important to assure that they are performed as part of an initial evaluation by a high-quality diagnostic center. Most diagnostic assessments are not repeated at each iteration but might be repeated at one or two key intervals during the long-term treatment course.

Other assessments can be obtained to examine and follow specific core ASD symptoms. For overall ASD symptoms, the Social Responsiveness Scale (SRS) can provide an overall index as well as symptom-specific indices, including those related to social interaction and repetitive behaviors and restricted interests, and the SRS corresponds well with the gold standards [31]. Repetitive behavior can also be measured using the Aberrant Behavior Checklist (ABC) [32]. In the recent DSM-5 criteria, sensory sensitivity has been added to the criteria, so an assessment of sensory sensitivities, such as the Sensory Profile 2 (SP2) questionnaire may be useful [33]. Since these are all caregiver-rated questionnaires, it is important to be aware of observer bias. 

Other assessments using objective observers would be optimal, but few such standardized measures exist and performing an extended objective evaluation not only burdens available resource but can provide inaccurate information, as patients often can only tolerate limited interactions. Other tests such as the Brief Observation of Social Communication Change (BOSCC) are also being developed to specifically examine change in ASD symptoms over time [34]. In our research studies, we have tried to efficiently combine resources by using the Structure Laboratory Observation test, which records interactions during a naturalist setting with the parent as well as a short examiner interaction [35,36]. We then had an objective observer to rate the videotaped interactions using the Ohio Autism Clinical Impressions Scale (OACIS) [37,38] which is a structure ASD symptom rating scale using in clinical trials [39]. 

Most importantly, the diagnosis of ASD requires that the symptoms significantly interfere with the individual life. Adaptative behavior scales such as the Vineland Adaptive Behavior Scale (VABS) or Adaptive Behavior Assessment System provide an index of the ability of the individual with ASD to function in everyday life [40].

### 3.2. Related Symptoms in Autism Spectrum Disorder

Many important ASD-related symptoms are important to assess and follow as they significantly impede function. Several psychiatric manifestations are important to assess. Irritability is such a significant symptom that the only approved medications for ASD specifically target this symptom. The ABC irritability scale is the standard assessment that defines this symptom [32]. Anxiety is a key symptom that impedes social interactions, which affects a significant portion of the ASD population [41]. The Parent-Rated Anxiety Scale for ASD [42] is a recently developed parent-rated questionnaire while the Screen for Child Anxiety Related Disorders (SCARED) has both child- and parent-rated questionnaires [41]. Attention Deficit Hyperactivity Disorder is highly comorbid with ASD and is known to modulate social responsiveness and competency [43] and can be assessed by the widely used standard no cost Vanderbilt questionnaire [44].

Executive dysfunction is prevalent in ASD, where it is associated with repetitive and restricted behaviors [45] and social competence [46] and can be a barrier to success as children with ASD transition into adulthood [47]. Simple questionnaires such as the Behavior Rating Inventory of Executive Function (BRIEF) can be useful as a general index but do not necessarily provide detailed information. Detailed confrontational testing may be necessary to uncover specific executive function processes that are disrupted.

More detailed analysis of language and intelligence may require detailed assessments. Both aspects are difficult to assess because of the wide range of abilities in the ASD population [48]. Assessment of intelligence is particularly difficult. Studies have suggested that intelligence quotient (IQ) is significantly underestimated by standard IQ evaluations due to the large language load of these tests and the frequent comorbid language abnormalities. Although specific non-verbal intelligence testing might provide a better estimate of IQ, some individuals with ASD also demonstrate non-verbal limitations. In general, ASD is associated with scattered skills which can vary widely which also make abbreviated IQ assessments prone to be inaccurate.

### 3.3. Evaluating Organ Specific Disorders in Autism Spectrum Disorder

Behavior is thought to be controlled by the brain, so it is no surprise that abnormalities in brain function are prevalent in children with ASD. Both structural and functional abnormalities in brain structure and connectivity can be found in some children with ASD [49,50]. Recent advances in neuroimaging have made it possible to not only investigate structural brain abnormalities but also to examine functional connectivity to detect cognitive pathway integrity [49] as well as neurotransmitter imbalances [51]. Additionally, the recent description of the potential for a locked-in network state in children with ASD suggests that resting-state functional neuroimaging may be helpful for prognosis of habilitation potential [49]. Of course, neuroimaging requires sedation, which has risks that need to be considered.

Sleep disruption, particular issues with sleep onset and maintenance, are particularly prevalent in ASD and can affect ongoing behavior during the day, including ASD symptoms [52]. The standard for sleep assessment in ASD, at least for eliciting symptoms, is the Childhood Sleep Habits Questionnaire [52], although a formal sleep study may be necessary for those with severe symptoms.

ASD is associated with epilepsy and subclinical electrical discharges (SEDs) which frequently occur during sleep and could indicate electrical status epilepticus during slow wave sleep [53,54]. Individuals with SEDs not uncommonly have ASD symptoms [55] and children with ASD appear to have a distinct frontal temporal characteristic pattern of SEDs [56]. Since epileptiform abnormalities may only be obvious during sleep, an overnight electroencephalograph is the preferred test [53].

One of the major advances in the field of ASD is the overlap between cerebral folate deficiency or insufficiency and ASD [57]. Indeed, individuals with cerebral folate deficiency clearly have ASD symptoms and individuals with ASD have an increased incidence of cerebral folate deficiency and insufficiency [57]. Although the gold-standard for measuring central folate levels is a lumbar puncture, the measurement of the folate receptor alpha autoantibody can be commercially obtained and is predictive of response to treatment for this disorder [58].

Individuals with ASD also commonly manifest motor and oculomotor apraxia which can be evaluated by an occupational therapist or eye specialist. Oculomotor performance may be particularly significant as this may result in impaired social interaction [59] and may impair focusing on visual targets and be related to repetitive behaviors [60,61].

GI disorders are extremely prevalent in ASD and they often go undiagnosed because children cannot communicate their symptoms. Although common symptoms such as constipation and feeding difficulties may be obvious, often more serious disorders such as gastroesophageal reflux and eosinophilic esophagitis may only manifest as repetitive behaviors or irritability [62]. Recently a brief parent-reported screen for common GI disorders has been developed for ASD [63].

Many immune disorders commonly occur in ASD [64]. Allergies, particularly food allergies [65], are not uncommon and can exacerbate ASD symptoms [64]. Immune deficiencies are not uncommon in ASD and biomarkers of immune system activation and suppression have been linked to ASD symptoms [66]. Autoimmune encephalitis with brain directed antibodies is also associated with ASD symptoms [67].

It is important to screen for endocrine disorders such as thyroid dysfunction in individuals with ASD, although many studies point to the key effect of thyroid disruption occurring prenatally as a result of environmental endocrine disruptors [68].

### 3.4. Evaluating Systemic Disorders in Autism Spectrum Disorder

Systematic disorders affect general cellular processes throughout the body, although they may manifest to a greater extent in one particular organ. For those with ASD, such disorders come in two categories: metabolic disorders and genetic disorders.

The most common metabolic disorders affecting those with ASD are mitochondrial disorders [69] and disorders of the transmethylation/transsulfuration pathways [70]. A wide variety of mitochondrial metabolism biomarkers have been described with many being associated with core and associated ASD symptoms [71]. For example, alternations in electron transport chain complex activity derived from buccal tissue [72] and fatty acid oxidation been linked to ASD-specific symptoms on the SRS [73] and variations in mitochondrial morphology have been linked to both ASD-specific symptoms on the SRS and core ASD symptoms on the ABC [74]. Abnormalities in transmethylation and transsulfuration spanning the methylation and redox pathways may be diagnostic for ASD [70] and have been shown to be related to neurodevelopment as indexed by the VABS in several studies [75,76]. Other, lesser studied, metabolic disorders have been described in ASD. Abnormalities in amino acid metabolism, particular dysregulation of branched chain amino acids, have been associated with a specific ASD phenotypes [77]. It should be mentioned that the biomarkers for metabolic disorders, in general, require expert interpretation by physicians knowledgeable in the field. As such, a specialist in metabolism should be involved in the multidisciplinary evaluation when a metabolic evaluation is conducted.

Common genetic abnormalities associated with ASD include copy number variations, CGG triplet expansion in the 5′-untranslated region of the fragile X mental retardation-1 gene, single gene and mitochondrial DNA mutations [78]. With advanced genetic techniques, genetic testing has been utilized to increasingly identify syndromes which were previously thought to be rare, such as Phelan-McDermid Syndrome and mTOR pathway-related disorders such as Tuberous Sclerosis Complex [79]. As more cases are identified, the specific characteristics of these syndromes with respect to ASD core and associated symptoms become better defined and will allow better management of these syndromes. Guidelines have recommended chromosomal microarray and Fragile X trinucleotide repeat testing as a first step, followed by whole exome sequencing if indicated [78]. However, some have pointed out that whole exome sequencing may be more cost-effective, especially in in consanguineous populations [80] and more advanced techniques such as whole genome sequencing and RNA sequencing are promising new tools [81].

### 3.5. Evaluating Environmental Influences in Autism Spectrum Disorder

Probably some of the most crucial factors to identify are the patient’s environment in the broadest sense, primarily because this may be easily and directly modifiable. Environmental factors include the everyday family, therapeutic and educational interactions with the child as well as what the child’s body is exposed to.

The home and family environmental psychosocial factors appear to be important in the outcomes of children with ASD. Conduct problems are related to socioeconomic factors in children with intellectual disabilities [82]. Most importantly, parental characteristics may be key to the child’s success, as studies have shown improved outcomes in parents with more psychological support [83], positive emotionality [84], engagement with the child [85] and in those that can accept the diagnosis and understand their child’s perspective [86]. Conversely, parental stress can adversely affect the child’s outcome [85], including causing them to have poorer self-regulation skills [87]. Appropriate instruments to measure these important aspects of life in children with ASD are not well developed. While many studies have conducted interviews or developed novel questionnaires, other studies have used the Parental Stress Index [87] or standardized quality-of-life questionnaires [10,12].

The school environment is key to providing the child with the proper services. An Individual Education Plan (IEP) should be standard, but its implementation can vary with many factors. IEPs should be reviewed by the child’s medical team and kept on file for review. The Autism Program Environment Rating Scale (APERS) is a validated instrument designed to assess the quality of ASD education programs [25]. All reports for therapies, such as speech, occupational, physical, music and/or behavior therapy, should also be reviewed.

Dietary and nutritional needs are of the utmost importance. First, feeding problems are pervasive in children with ASD. Such patterns can be cause by GI issues such as gastroesophageal reflux [62], avoidant/restrictive food intake disorder [88] and/or food allergies causing GI inflammation such as eosinophilic esophagitis [89]. Second, children with ASD are known to be susceptible to vitamin and micronutrient deficiencies, including vitamin D deficiency and insufficiency [90], vitamin A, cobalamin, zinc and iron deficiency [91], which can sometimes be secondary to avoidant/restrictive food intake disorder [92] and can be related to ASD severity [91]. Thus, the evaluation of the dietary intake is twofold, both to ensure proper nutrition is being consumed and to assure that the child is not being exposed to substances that they might be allergic or sensitive to. Selected vitamin levels are wise to obtain, especially if feeding issues exist.

ASD is associated with food allergies [93] and hypersensitivity [65] and atopic dermatitis [94] with at least one study suggesting a relationship between these atopic disorders and communication development [95]. Persistent atopic dermatitis with emergent atopic respiratory disease is also related to ASD [96]. Many feeding problems in children with ASD are related to food allergies and tactile hypersensitivity. Thus, atopic disease is an important disorder to screen for in children with ASD and treat, as such disorders can cause significant discomfort, reduce intake of essential nutrients and may exacerbate ASD symptoms.

Environmental toxins including air pollution, phthalate and pesticides are associated with ASD [14]. The physiological effect of environmental toxins includes endocrine disruption, mitochondrial dysfunction, inflammation and increased oxidative stress. These latter three processes are already increased in ASD, so it is possible that children with ASD may be less resilient to these common environmental agents, resulting in increased symptomology.

### 3.6. Potential Treatable Disorders in Autism Spectrum Disorder

Health issues in children with ASD are associated with lower quality of life [12] and more health problems [10] in the parents through possible spillover effects. Improving health problems, such as sleep disorders, may improve the quality of life [12] and psychosocial stress [97] of the parents and family. Thus, the health and symptoms of children with ASD may be substantially improved by addressing these issues (See Table 3).

Behavior and psychiatric symptoms can be mitigated in several ways. Irritability may respond to parental training programs [98,99]. Treatment of anxiety in children with ASD is somewhat uncertain. Standard treatments with selective serotonin reuptake inhibitors result in variable outcomes and may do more harm than good [100], while cognitive behavioral therapy appears to be superior to standard treatments [101]. Other promising safe medications such as propranolol [102] and novel methodologies such as transdermal electrical neuromodulation [41] have also shown some promising outcomes for anxiety. Treatment of attention deficit hyperactivity disorder is complicated as there is a greater adverse effect burden with standard stimulant medication in children with ASD so alpha-adrenergic medications may be a better choice [103].

Individuals with epilepsy and ASD should be treated with anti-epileptic medications [53,54] and receive a workup for underlying genetic and metabolic disorders [53,104]. Some children with ASD and underlying epileptiform abnormalities may show improvement in symptoms with anti-epileptic medication [55,105]. Sleep is of the utmost importance and a stepwise treatment protocol starting with good sleep hygiene with escalation to melatonin and then other pharmacologic or supplement treatments should be pursued [52]. Treatments for cerebral folate abnormalities include leucovorin as well as a milk-free diet [58]. Treatments for GI disorders in children with ASD have been outlined by an expert panel [62] and prebiotics and probiotics may be effective [106]. Immune disorders vary widely: atopic disease should be addressed either with elimination of the allergen or with medication or referral of an allergist while autoimmune disorders and immunodeficiencies may respond to IVIG but should be evaluated and treated by an immunologist [66].

Treatment of nutritional deficiencies can be straightforward, while implementing dietary changes can be complicated by behavioral problems [28]. Preliminary studies suggest that vitamin D [90] and/or zinc [107] supplementation might improve ASD symptoms. Treatment of metabolic disorders can be complex [108] and should be managed by a specialist. Abnormalities in methylation metabolism have been shown to respond to methylcobalamin [108] while treatments of redox metabolism may respond to either methylcobalamin [108] or N-acetylcysteine (NAC) [109].

School interventions outlined in an IEP are necessary for a comprehensive education program, but unfortunately recent studies show that schools do not consistently meet standard outlined by federal law or best practice recommendations [110]. The school districts approach to implementing ASD-specific programs may have a significant effect on the child’s outcomes. School districts with proactive implementation of interventions tend to use evidence-based practices and develop ASD-specific programs, whereas school districts with reactive implementation of programs tend to have more litigation and due process and greater escalation of student behavior [111]. Some school districts develop comprehensive programs specifically for children with ASD [112]. If possible, inclusion in mainstream schools may have positive outcomes for some children with ASD [113]. The sensory environment at school can be overwhelming, leading to disruptive behaviors. Standard treatment should include occupational therapy based sensory integration therapy, a sensory diet, and environmental modifications [27,114].

## 4. Implementing a Comprehensive Assessment in the Clinical Setting

**Figure 3:**
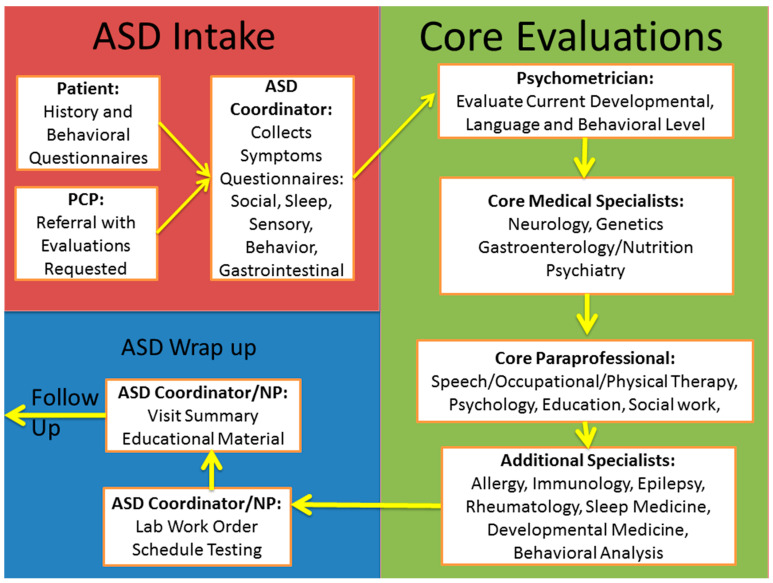
Overview of the workflow through a personalized multispecialty medical clinic to comprehensively evaluate children with ASD in a personalized manner. Multiple specialists and paraprofessionals are needed to identify treatable abnormalities associated with ASD.

Figure 3 outlines an example of the implementation of a comprehensive assessment and treatment plan within the context of the medical clinic. A structured and comprehensive intake process should be followed by objective assessment by a psychologist or psychometrist. A core set of specialists and paraprofessionals are needed to perform a comprehensive evaluation, especially on the first visit. Depending on the individual patient, additional specialists and paraprofessional should be consulted. Once a comprehensive plan is assembled, testing and educational material should be provided to the family. Follow-up needs to be coordinated to continue to apply the most promising treatments and optimize the child’s health.

## 5. Discussion

ASD is a prevalent and life-long neurodevelopmental disorder with no known cure. Identifying and targeting underlying medical conditions, behavioral abnormalities and adverse environmental influences have the potential to significantly improve the quality of life for children with ASD and their families. A systematic approach to evaluating and treating these related disorders is important. Minimizing core and associated ASD symptoms could substantially improve the lives of individuals with ASD. Decreasing the influence of such symptoms on an individual’s functional ability to the point that they no longer require support would practically and diagnostically remove the ASD diagnosis.

Further research will be needed to better understand the optimal method for identifying underlying disorders and prioritizing treatment. Although there are many promising biomarkers for pathophysiological processes associated with ASD, many are not yet diagnostic because of a lack of validation clinical studies. Furthermore, the exact nature of associated abnormalities is still not well defined. For example, many children with ASD have abnormalities in sleep maintenance, but the exact underlying biological processes that cause such issues are still not well defined. As a corollary, optimal treatments for associated disorders are not well studied. Although treatments can be borrowed from other areas of medicine, children with ASD tend to have idiosyncratic response to many treatments, making their management complicated, and further solidifying the idea of a systematic evidence-based stepwise approach. It is important for physicians and paraprofessionals to be aware of this approach.

## 6. Conclusions

By identifying medical and environmental factors associated with ASD that can modulate symptoms, a targeted systematic approach can be developed to address such factors in order to improve the lives of children with ASD and their families. The approach outlined here is preliminary but has the potential to be developed to provide substantial benefit to many with ASD.

## Figures and Tables

**Table 1:** Diagnostic criteria for Autism Spectrum Disorder as well as Specifiers and common associated behavioral and cognitive symptoms.

Core Autism Symptoms	Specifiers	Associated Symptoms
Social Communication Impairment ^1^	Intellectual impairment	Irritability
Social-emotional reciprocity	Language impairment	Impulsivity
Nonverbal communication	Medical condition	Anxiety
Understand relationships	Genetic condition	Attention Deficit
Restricted, Repetitive Behavior ^1^	Environmental factors	Hyperactivity
Repetitive motor movements	Other DSM diagnosis	Executive Dysfunction
Inflexible to change	Catatonia	Learning Disorder
Restricted, fixated interests		
Sensory Sensitivities		

^1^ The severity of each of these two core domains are rated based on the amount of support required: (1) Requiring support, (2) Requiring substantial support, or (3) Requiring very substantial support.

**Table 2:** Measurement tools used to assess the severity of common disorders and their associated symptoms in children with Autism Spectrum Disorder.

Disorder Domain	Example	Potential Measurement Instruments
	**Core Autism Symptoms**	
Social Communication	Social Function	ADOS, ADI-R, Social Responsiveness Scale
Repetitive Behavior	Stereotypies	ADOS, ADI-R, Aberrant Behavior Checklist
Sensory Sensitivities	Noise Sensitivity	Sensory Profile 2 Questionnaire
Adaptive Behavior	Daily Living Skills	Vineland Adaptive Behavior Scale
	**Autism Related Symptoms**	
Psychiatric Manifestations	Irritability	Aberrant Behavior Checklist
	Anxiety	PRAS-ASD Questionnaire
	Attention Deficit Hyperactivity Disorder	Vanderbilt Questionnaire
Executive Dysfunction	Cognitive Perseveration	BRIEF Questionnaire
Cognitive Function	Intelligence	Intelligence Testing
Communication	Language	Language Assessment
	**Organ Specific Disorders**	
Brain	Structural Brain Abnormalizes	Structural MRI
	Locked-in Network State	Resting State Functional MRI
	Epileptiform Abnormalities	Overnight EEG
	Sleep Disruption	Childhood Sleep Habits Questionnaire
	Cerebral Folate Insufficiency	Folate Receptor Autoantibody
	Apraxia	Occupational Therapy Evaluation
Gastrointestinal	Constipation	Brief parent-report screen for common GI disorders
	Gastroesophageal Reflux	Gastroenterology Evaluation
	Eosinophilic Esophagitis	Gastroenterology Evaluation
Immune Disorders	Atopic Disorders	Allergist Evaluation
	Autoimmune Disorders	Rheumatology Evaluation
	Immunodeficiency	Immunology Evaluation
Endocrine Disorders	Thyroid Abnormalities	Thyroid Stimulating Hormone & Panel
	**Systemic Disorders**	
Metabolic Disorders	Mitochondrial Dysfunction	Fasting Mitochondrial Labs
	Fatty Acid Oxidation Defects	Carnitine/Acyl-Carnitine Panels
	Amino Acid Metabolism	Serum/Plasma Amino Acids
	Urea Cycle Defects	Ammonia & Amino Acids
	Purine Metabolism Defects	Uric Acid
Methylation Metabolism	Low S-Adenosylmethionine (SAM)	Fasting Homocysteine
Redox Metabolism	Low Glutathione	Free and Total Glutathione
Genetic Disorders	Chromosomal Abnormality	Chromosomal Microarray
	Fragile X	CGG Repeats in FMR1 gene
	Single Gene Mutation	Whole Exome Sequencing
	mtDNA Mutation	mtDNA Sequencing
	**Environmental Factors**	
Psychosocial	Family Stress and Coping	Parental Stress Index, CarerQol
Educational	Therapies	Therapy Report
	School Interventions	Autism Program Environment Rating Scale
Nutritional	Vitamin D Deficiency	Vitamin D Level
	Co-Enzyme Q10 Deficiency	CoQ10 Blood Level
	Zinc Deficiency	Zinc Level
	Iron Deficiency	Serum Ferritin
Allergens	Animal/Seasonal Allergy	Allergy Exposure Questionnaire
Toxicants	Chemical Sensitivity	QEESI Questionnaire
	Air Quality	EPA Air Quality Monitor

**Table 3:** Examples of Potentially Treatable Conditions Associated with Autism Spectrum Disorder.

Factor	Example	Potential Intervention
**Autism Related Symptoms**		
Psychiatric	Irritability	Parent Training Program
	Anxiety	Cognitive Behavioral Therapy
	Attention Deficit Hyperactivity Disorder	Alpha-Adrenergic Medications
**Organ Specific Disorders**		
Brain	Epileptiform Abnormalities	Anti-epileptic Medications
	Sleep Disruption	Sleep Hygiene, Melatonin
	Cerebral Folate Insufficiency	Leucovorin Calcium
Gastrointestinal Disorders	Constipation	Magnesium
	Gastroesophageal Reflux	Proton Pump Inhibitor
	Eosinophilic Esophagitis	Food Allergen Elimination
Immune Disorders	Atopic Disorders	Allergen Elimination
	Autoimmune Encephalopathy	IVIG, Immunomodulation
	Immunodeficiency	IVIG
**Systemic Disorders**		
Metabolic Disorders	Mitochondrial Dysfunction	Mitochondrial Cocktail
	Carnitine Deficiency	L-Carnitine
Nutritional Disorders	Vitamin D Deficiency	Vitamin D
	Zinc Deficiency	Zinc Supplementation
Redox Metabolism	Low Glutathione	N-Acetylcysteine
Methylation Metabolism	Low Homocysteine	Methylcobalamin
Genetic Disorders	Down Syndrome	Anticipatory Surveillance
	**Environmental Factors**	
Psychosocial	Family Stress	Social Work
	Family Coping	Psychological Support
	Quality of Life	Improving Child Health
Educational	Therapies	Disability Coordinator
	Classroom	Individual Education Plan
Sensory	Sensory Overload	Sensory Diet
